# IFN-γ, IL-4 and IL-13 modulate responsiveness of human airway smooth muscle cells to IL-13

**DOI:** 10.1186/1465-9921-9-84

**Published:** 2008-12-30

**Authors:** Barry J Moynihan, Barbara Tolloczko, Souad El Bassam, Pascale Ferraro, Marie-Claire Michoud, James G Martin, Sophie Laberge

**Affiliations:** 1 The Meakins-Christie Laboratories, Department of Medicine, McGill University, Montreal, Canada; 2 The Department of Surgery, University of Montreal, Montreal, Canada; 3 Respiratory Medicine Division, CHU Sainte-Justine, Department of Pediatrics, University of Montreal, Montreal, Canada

## Abstract

**Background:**

IL-13 is a critical mediator of allergic asthma and associated airway hyperresponsiveness. IL-13 acts through a receptor complex comprised of IL-13Rα1 and IL-4Rα subunits with subsequent activation of signal transducer and activator of transcription 6 (STAT6). The IL-13Rα2 receptor may act as a decoy receptor. In human airway smooth muscle (HASM) cells, IL-13 enhances cellular proliferation, calcium responses to agonists and induces eotaxin production. We investigated the effects of pre-treatment with IL-4, IL-13 and IFN-γ on the responses of HASM cells to IL-13.

**Methods:**

Cultured HASM were examined for expression of IL-13 receptor subunits using polymerase chain reaction, immunofluorescence microscopy and flow cytometry. Effects of cytokine pre-treatment on IL-13-induced cell responses were assessed by looking at STAT6 phosphorylation using Western blot, eotaxin secretion and calcium responses to histamine.

**Results:**

IL-13Rα1, IL-4Rα and IL-13Rα2 subunits were expressed on HASM cells. IL-13 induced phosphorylation of STAT6 which reached a maximum by 30 minutes. Pre-treatment with IL-4, IL-13 and, to a lesser degree, IFN-γ reduced peak STAT6 phosphorylation in response to IL-13. IL-13, but not IFN-γ, pre-treatment abrogated IL-13-induced eotaxin secretion. Pre-treatment with IL-4 or IL-13 abrogated IL-13-induced augmentation of the calcium transient evoked by histamine. Cytokine pre-treatment did not affect expression of IL-13Rα1 and IL-4Rα but increased expression of IL-13Rα2. An anti-IL-13Rα2 neutralizing antibody did not prevent the cytokine pre-treatment effects on STAT6 phosphorylation. Cytokine pre-treatment increased SOCS-1, but not SOCS-3, mRNA expression which was not associated with significant increases in protein expression.

**Conclusion:**

Pre-treatment with IL-4 and IL-13, but not IFN-γ, induced desensitization of the HASM cells to IL-13 as measured by eotaxin secretion and calcium transients to histamine. The mechanism of IL-4 and IL-13 induced desensitization does not appear to involve either downregulation of receptor expression or induction of the IL-13Rα2 or the SOCS proteins.

## Background

Asthma and allergy have been associated with a Th2 polarized immunological response to otherwise innocuous allergens. This Th2 response is associated with increased levels of the interleukins IL-4 and IL-13 in inflamed tissues [1-3]. The Th2 paradigm has been successfully reproduced in animal models of allergen sensitization and challenge to antigens such as ovalbumin [4-7]. Animal models demonstrate a role for IL-13 in the development of airway hyperresponsiveness (AHR); overexpression of IL-13 in the murine lung induces a phenotype similar to human asthma, with excess mucus production, goblet cell hyperplasia, smooth muscle hypertrophy and AHR [8,9]. IL-13 and IL-4/IL-13 knockout mice are protected from allergen induced AHR [5,10]. Interferon-γ may counteract the actions of IL-4 and IL-13 in some circumstances. IFN-γ knockout mice have been shown to have augmented Th2 responses and IFN-γ knockout mice have enhanced AHR following allergen challenge that is restored by administration of recombinant IFN-γ [11].

IL-13 and IL-4 signal through binding to their respective subunit of a receptor complex comprised of the IL-13 receptor alpha1 (IL-13Rα1) and the IL-4 receptor alpha (IL-4Rα) with subsequent phosphorylation of Janus activated kinases (JAKs) and the transcription factor signal transducer and activator of transcription 6 (STAT6) [12]. These pathways appear to be essential for the development of AHR as IL-4Rα and STAT6 deficient mice are protected from AHR [13-15] and reconstitution of STAT6 in the epithelium has been shown to be sufficient to restore IL-13 mediated AHR [16]. IL-13 also binds to the IL-13 receptor alpha2 (IL-13Rα2) with high affinity. The functional significance of this subunit is unclear. It may act as a decoy receptor to prevent excessive IL-13 signaling as it has a short intracytoplasmic tail [17-20] but there is also some evidence that it participates in IL-13 signaling [21]. IL-4 can drive many of the same processes as IL-13 through their shared receptor complex although IL-4 deficient mice are not protected from AHR [22].

Recent evidence indicates that cytokine actions on airway smooth muscle may be important in the pathobiology of asthma. The calcium responses to histamine, bradykinin and methacholine in human airway smooth muscle (HASM) cells are augmented by IL-13 [23]. The proliferative response of HASM cells to leukotriene D_4 _(LTD_4_) is also augmented by IL-13 [24]. The expression of the chemokines eotaxin, eotaxin-3 and TARC is also induced by IL-13, so IL-13 can be linked to the contractile, proliferative and secretory properties of HASM cells [25-27]. IFN-γ and IL-13 both increase HASM cell stiffness as assessed by magnetic twisting cytometry and reduce relaxation responses induced by isoproterenol [28]. We hypothesised that the Th2 cytokines IL-4 and IL-13 would have different effects than the Th1 cytokine IFN-γ on IL-13 signaling in HASM cells. We chose to use STAT6 phosphorylation, eotaxin production and calcium responses as outcomes for IL-13 signaling. ASM is a primary effector of bronchoconstriction and IL-13 increases the contractility of HASM cells [23] and promotes STAT6-dependent eotaxin production [29].

## Methods

### Cell cultures

Primary cultures of HASM cells were prepared from lung transplant specimens as previously described [30]. Briefly, tissue digestion was obtained by incubating the tissues in HBSS containing collagenase type IV (0.4 mg/ml), elastase (0.38 mg/ml) and soybean trypsin inhibitor (1 mg/ml) at 37°C for 90 min with gentle shaking. The dissociated cells were collected by filtering through 125 μm Nytex mesh followed by centrifugation. The pellet was then reconstituted in growth medium (DMEM-Ham's F12 medium supplemented with 10% fetal bovine serum, penicillin 10000 unit/ml, streptomycin 10 mg/ml, and amphotericin 25 μg/ml) and plated in 25-cm^2 ^flask. Confluent cells were detached with a 0.025% trypsin solution containing 0.02% ethylenediaminetetraacetic acid (EDTA). Cells were grown on glass cover slips for single immunofluorescence microscopy and calcium measurements or on 6 well culture dishes for flow cytometric analysis and for protein and mRNA extraction. Confluent cultures of HASM cells were serum deprived and supplemented with 0.1% bovine serum albumin, 5 μg/ml insulin and 5 μg/ml transferrin 24 h prior to cytokine treatment. Cells at passages 3–6 were used in all experiments.

### Immunofluorescence microscopy

Serum-starved human HASM cells were cultured to semiconfluence on coverslips, washed with PBS, fixed for 20 min at RT in 4% formaldehyde in PBS and incubated for 1 hour with 10% FBS in PBS before the addition of the primary antibody. The cells were then incubated for 1 hour with the following primary antibodies used at the appropriate dilution: anti-IL-13Rα1 mAb (CD213a1, Diaclone, Besançon, France), anti-IL-13Rα2 (CD213a2, Diaclone, Besançon, France), anti-IL-4Rα (CD124, Pharmingen, BD Biosciences, Mississauga, Canada). These primary antibodies were detected with Cy2-conjugated goat anti-mouse IgG (dilution 1/50). Slides were washed twice with PBS and counterstained with nuclei stain DAPI for 5 min. As controls, cells were processed either in the absence of the primary antibody or using an isotype control. After washing, coverslips were mounted on glass slides, visualized with a Zeiss Axioskop 2 microscope and photographs were taken using a SPOT camera with SPOT RT software (SPOT Diagnostic Instruments).

### Flow cytometry analysis

Cells were treated with IL-4, IL-13 or IFN-γ at various concentrations for 24 h. Cells were washed with PBS, detached with a non-enzymatic cell dissociation buffer and then washed with staining buffer (Dulbecco's PBS, 1% heat-inactivated FBS, 0.09% sodium azide) and centrifuged. After blocking Fc receptors, cells were incubated for 3 h on ice with either anti-IL-13Rα1-PE (Diaclone, Besançon, France), anti-IL-13Rα2-PE (Diaclone, Besançon, France), or anti-IL-4Rα-FITC (Pharmingen, BD Biosciences) monoclonal antibodies. Isotype-matched mouse antibodies were used in all experiments. Cells were fixed in 2% paraformaldehyde. Flow cytometric acquisition and analysis of samples were performed on at least 10,000 acquired events on a FACScan (Becton-Dickinson, Oakville, Canada). Dead cells were excluded by gating out propidium iodide-positive cells during assessment of cytokine receptor surface expression. Analysis was performed using Cell Quest software (BD Biosciences).

### RNA isolation and real-time RT-PCR

Total RNA was isolated from HASM cells using TRIzol reagent (Invitrogen, Carlsbad, CA), according to the manufacturer's instructions. Extracted total RNA was dissolved in RNA-free water, quantified at 260 nm and 100 ng of total cellular RNA was used for analysis. One-Step quantitative real-time RT-PCR was conducted using the QuantiTect SYBR Green System (Qiagen, Mississauga, Canada) on a Mx3000P cycler (Stratagene, La Jolla, CA). The sequences of the specific primer pairs that were used for each gene of interest are displayed in Table 1; specific primers were used at a final concentration of 1 μM. The reverse transcription was done at 50° for 30 minutes. Cycling conditions were one cycle at 95°C for 15 min, followed by 45 cycles of denaturation at 94° for 15 s, annealing at 60° for 30 s and extension at 72° for 30 s. Melting curve analysis was used to document the amplicon specificity. Calibration curves were generated by measuring serial dilutions of stock cDNA to calculate the amplification efficiency. The relative amount of mRNA for each target gene was normalized to the value obtained for the housekeeping gene 18S.

**Table 1:** Primers used for real-time polymerase chain reaction analysis of gene expression.

**Gene**	**Forward primer (5' to 3')**	**Reverse primer (5' to 3')**	**Product length (bp)**
IL-13Rα1	atctcacccccagaaggtgat	cgggactggtattccttc	119

IL-13Rα2	cctttgccgccagtctatctta	tcaaaacaccttgctggaatagg	108

IL-4Rα	gctatgtcagcatcaccaagattaa	cccctgagcatcctggattat	101

SOCS-1	ggaactgctttttcgccctta	agcagctcgaagaggcagtc	127

SOCS-3	gtccccccagaagagcctatta	ttgacggtcttccgacagagat	118

### Protein extraction and Western Blotting

Confluent cultures of HASM cells were serum starved as above. After stimulation, cells were washed with ice-cold PBS and lysed. Samples were sonicated, boiled and protein content was quantified using Bradford assay. Equal quantities of protein were loaded per lane. Electrophoresis was carried out by using 8% (Phospho-STAT6, STAT6, rabbit polyclonal IgG, dilution 1:1000, UpState, Lake Placid, NY, USA; Phospho-ERK, ERK, rabbit polyclonal IgG, dilution 1:1000, Cell Signaling Technology) or 15% (rabbit polyclonal anti-SOCS-1 IgG, dilution 1:1000; mouse monoclonal anti-SOCS-3 IgG, dilution 1:1000, Abcam Inc, Cambridge, MA) SDS-PAGE gel and proteins were transferred to nitrocellulose membranes. Membranes were blocked for 1 hour at room temperature for STAT6 with 5% dried milk in Tris-HCl containing Tween 20 (TTBS) or 1% BSA with EDTA and NaCl in TTBS for SOCS-1 and SOCS-3. The membranes were incubated with primary antibodies overnight at 4°C followed by incubation with secondary antibodies (goat anti-rabbit IgG HRP conjugated, 1:1500, Upstate, or donkey anti-mouse IgG HRP conjugated, Jackson Immunology, dilution 1:10000, as appropriate) at room temperature for 1 hr. Blots were developed by chemiluminescence and the signals were recorded with Fluoro™ 800 Advanced Fluorescence Imager (Alpha Innotech Corporation, Montreal, Qc, Canada) and densitometry was performed with Fluorochem™ software. Negative controls without primary antibody were performed for all primary antibodies. Blots for β-actin, STAT6 and ERK were performed in parallel to verify comparable protein loading.

### ELISA

Eotaxin release in supernatants was analysed by means of ELISA (R&D Systems, Minneapolis, MN) according to the manufacturer's instructions. Every measurement was performed in duplicate.

### Measurement of intracellular free Ca^2+^

HASM cells grown on 25 mm diameter coverslips were used 10–14 days post plating. Cells were incubated for 30 min at 37°C with Hanks' buffer (in mM: NaCl 137, NaHCO_3 _4.2, glucose 10, Na_2_HPO_4 _3, KCl 5.4, KH_2_PO_4 _0.4, CaCl_2 _1.3, MgCl_2 _0.5, MgSO_4_0.8, *N*-2-hydroxyethylpiperazine-*N*'-ethane sulfonic acid [Hepes] 5) containing 5 μM Fura-acetoxymethylester (Fura-2-AM) and 0.02% pluronic F-127. The loaded cells were then washed and the coverslips placed in a Leiden chamber (Medical Systems Corp, Greenville, NY) containing 450 μL of Hanks' buffer on the stage of an inverted microscope equipped for cell imaging with a 40× oil objective (Nikon Corp, Tokyo, Japan). The cells were imaged using an intensified camera (Videoscope IC 200) and PTI software (Photon Technology International Inc, Princeton, NJ) at a single emission wavelength (510 nm) with double excitatory wavelengths (345 and 380 nm). The fluorescence ratio (345/380) was measured in individual cells (n = 8 per slide) and the free [Ca^2+^]_i _calculated according to Grynkiewicz's formula [31] using a k.d of Ca^2+ ^to Fura-2 of 224 nM R_max _was determined in cells exposed to ionomycin 10^-5 ^M in the presence of 1.3 mM CaCl_2 _and R_min _in Ca^2+ ^free buffer to which EGTA 10^-3 ^M and ionomycin 10^-5 ^M had been added. Background fluorescence and autofluorescence were automatically subtracted.

### Statistical analysis

Results are expressed as means and standard deviation values. The statistical analysis was performed using one-way ANOVA for repeated measurements followed by the Dunnett test for multiple comparisons. Statistical significance was established at a 5% level of confidence.

## Results

### HASM cells express IL-13 receptor subunits and IL-4, IL-13 and IFN-γ inhibit STAT6 phosphorylation induced by IL-13

**Figure 1:**
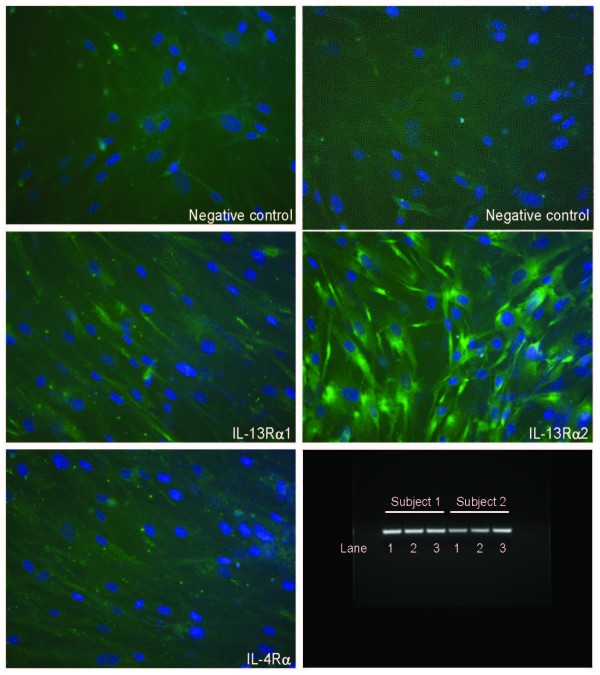
**Expression of IL-13 receptor subunits in cultured HASM**. The following primary antibodies were used: anti-IL-13Rα1 mAb, anti-IL-13Rα2 mAb and anti-IL-4Rα mAb. These primary antibodies were detected with Cy2-conjugated goat anti-mouse IgG (green). Slides were counterstained with nuclei stain DAPI (blue). As control, cells were processed in the absence of the primary antibody and/or the secondary antibody. mRNA transcripts for all subunits in HASM cells obtained from two different subjects are also shown: lane 1: IL-13Rα1; lane 2: IL-13Rα2; lane 3: IL-4Rα.

**Figure 2:**
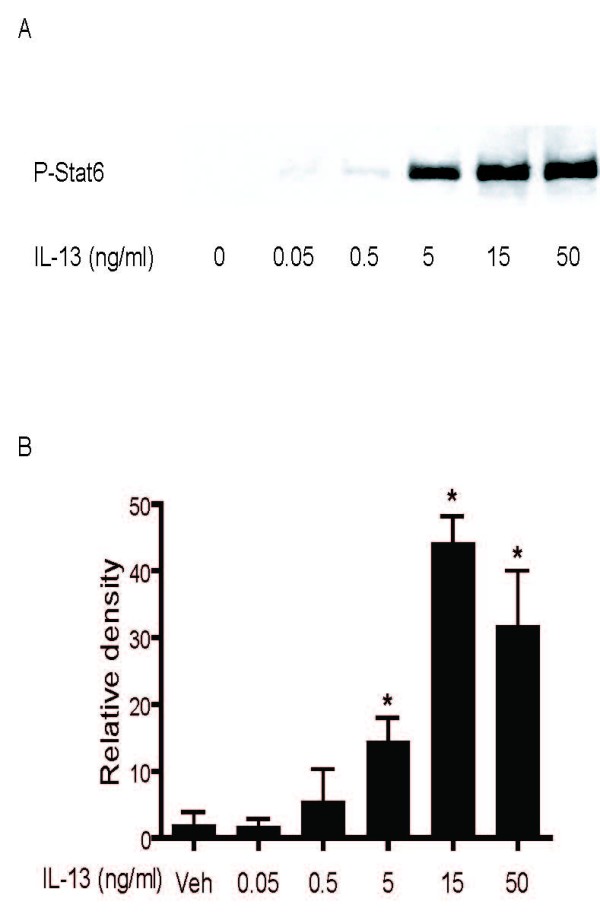
**Concentration-dependent effect of IL-13 on STAT6 phosphorylation**. HASM were stimulated with IL-13 for 30 minutes. Levels of phosphorylated STAT6 in cell lysates were detected by Western blot analysis using antibody that recognizes the tyrosine-phosphorylated forms of STAT6. A: representative Western blot film. B: Detected phosphorylated STAT6 band densities were measured and densitometric values presented as mean values ± SEM of two independent experiments.

The expression of the IL-13 receptor subunits in HASM cells was examined using RT-PCR and immunofluorescence microscopy. RNA preparation from cultured HASM cells revealed mRNA expression of the IL-13Rα1 subunit, the IL-13Rα2 subunit and the IL-4Rα subunit (Figure 1). We confirmed the expression of all the subunits by immunofluorescence microscopy (Figure 1). The IL-13 receptor complex was functional as evidenced by STAT6 phosphorylation following stimulation with IL-13 which reached a maximum by 30 minutes after stimulation. A dose-response curve of STAT6 phosphorylation showed that the peak response to IL-13 was observed at a concentration of 15 ng/ml (Figure 2). We chose the submaximal concentration of 5 ng/ml of IL-13 for all subsequent experiments investigating the effects of cytokine pre-treatment on IL-13 receptor activation.

**Figure 3:**
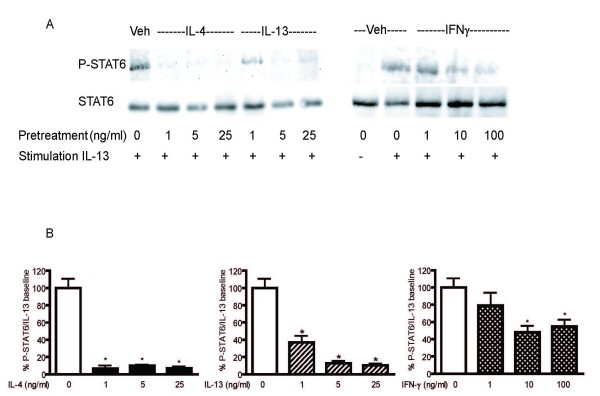
**Effects of IL-4, IL-13 and IFN-γ pre-treatment in STAT6 phosphorylation and total STAT6 protein induced by IL-13**. HASM cells were pre-treated with various concentrations of IL-4 (n = 3), IL-13 (n = 3) or IFN-γ (n = 5) for 24 hours and then stimulated with IL-13 (5 ng/ml) for 30 minutes. Whole cell lysates were extracted and subjected to Western analysis using antibodies that recognize total and phosphorylated forms of STAT6. A: Representative Western blot films. B. Detected phosphorylated – STAT6 band densities were measured and the densitometric values were normalized to the IL-13-stimulated value. Data are shown as the mean values ± SEM of 3–5 independent experiments. * p < 0.05 compared to IL-13 stimulated value in the absence of cytokine pre-treatment.

To assess the effects of Th2 and Th1 cytokines on IL-13 receptor activation, HASM cells were treated with increasing concentrations of IL-4, IL-13 and IFN-γ for 24 hours prior to IL-13 stimulation. No wash was performed prior to IL-13 stimulation in order to better reflect the *in vivo *state by a continuous exposure to cytokines in the culture milieu. Pre-treatment with all three cytokines significantly reduced peak STAT6 phosphorylation in response to IL-13 which was not associated with reduced total STAT6 expression (Figure 3). The magnitude of the effects of IL-4 and IL-13 pre-treatment on STAT6 phosphorylation induced by IL-13 was higher than that seen with IFN-γ pre-treatment. From these experiments, we chose to use the cytokines at the following concentrations for all subsequent experiments: IL-4 and IL-13 (5 ng/ml) and IFN-γ (10 ng/ml). The effects of cytokine pre-treatment on phosphorylation of ERK, another kinase associated with IL-13 receptor activation, was also studied. Cytokine pre-treatment with either IL-4, IL-13 or IFN- γ did not alter IL-13-induced ERK phosphorylation (data not shown).

### IL-4, IL-13 and IFN-γ increase expression of IL-13Rα2 in human airway smooth muscle cells

**Figure 4:**
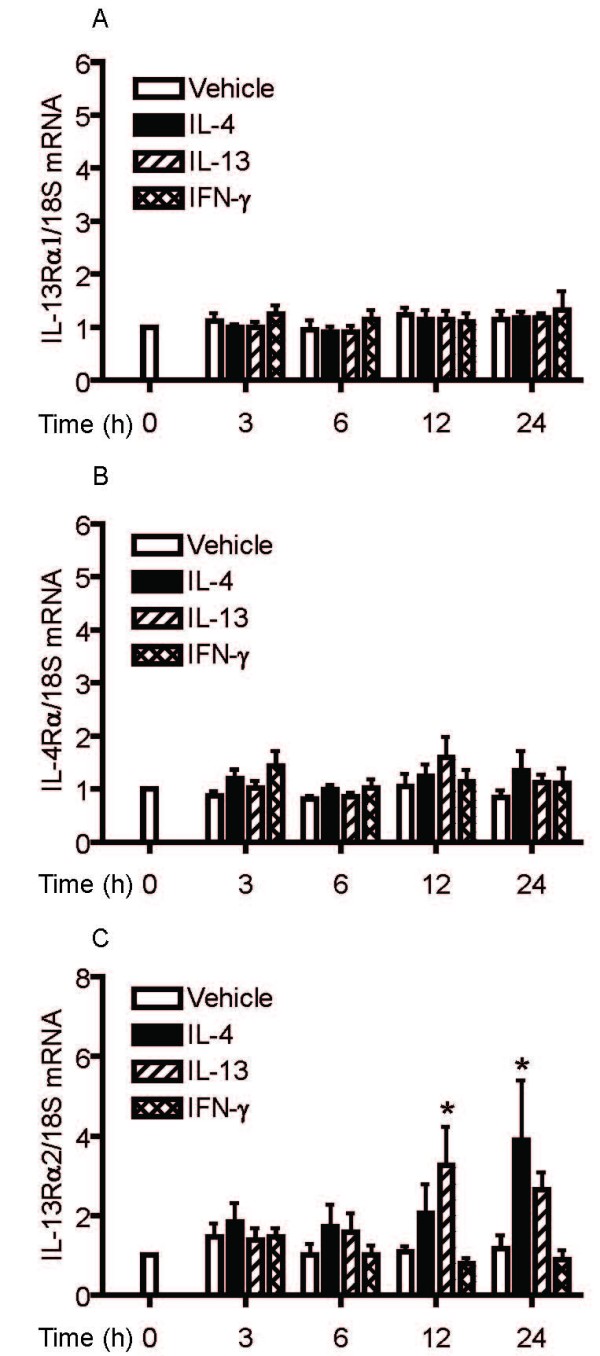
**Time-dependent effects of IL-4, IL-13 and IFN-γ on IL-13Rα1 (A), IL-4Rα (B) and IL-13Rα2 (C) mRNA expression**. HASM were incubated with vehicle, IL-4 (5 ng/ml), IL-13 (5 ng/ml) or IFN-γ (10 ng/ml) for 3, 6, 12 and 24 hours. Receptor subtype and 18S mRNA expression was quantified using real-time PCR. Data are expressed as the relative expression in receptor subtype/18S mRNA ratio and compared to unstimulated (vehicle) value of 1 at time 0, for five independent experiments. * p < 0.05 compared to cells treated with vehicle (ANOVA).

**Figure 5:**
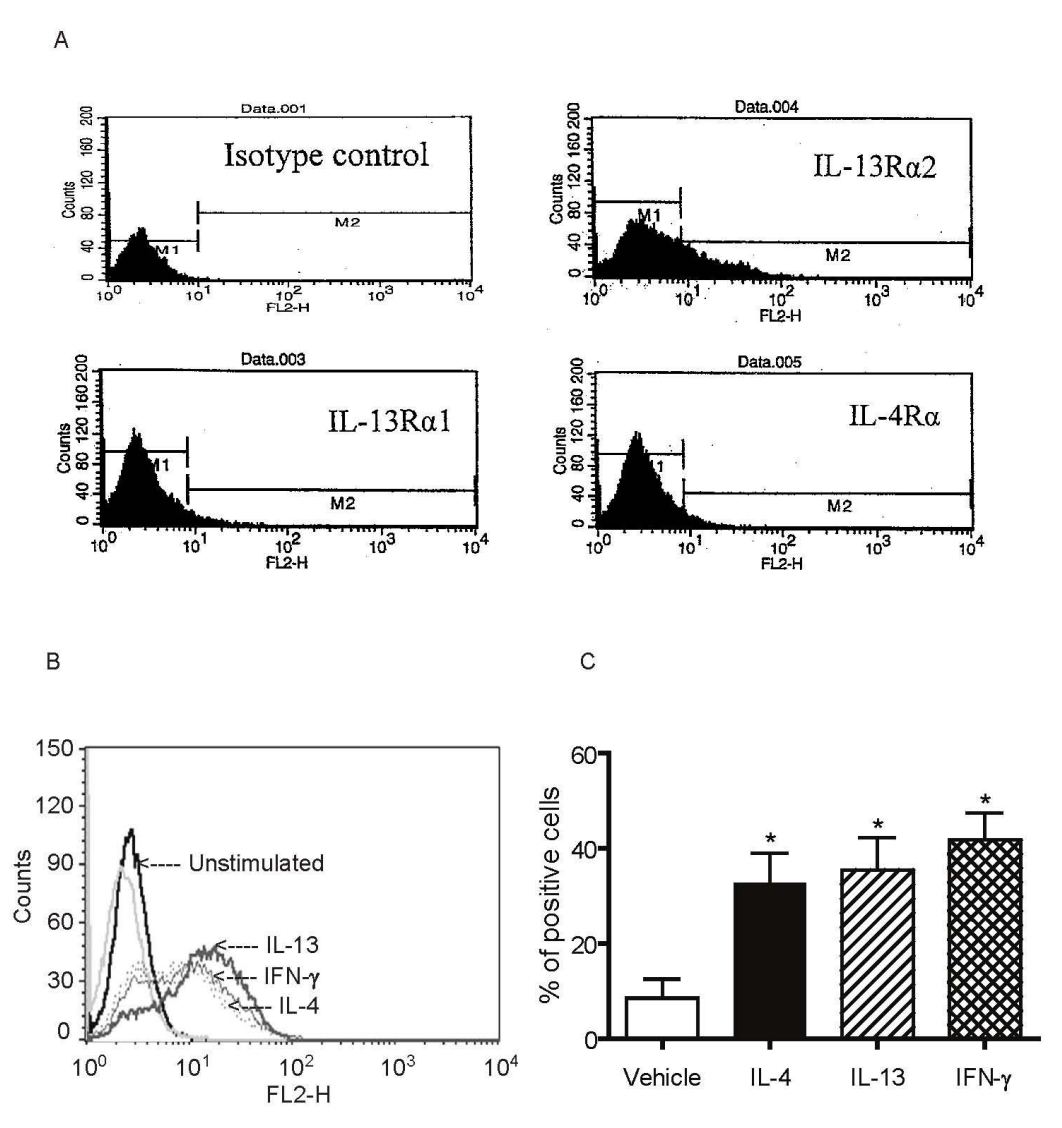
**Effects of IL-4, IL-13 and IFN-γ on cell surface expression of IL-13α2 receptor**. A. Representative flow cytometric analysis of IL-13 receptor subunits expression in resting cells. B: Representative flow cytometric analysis of IL-13Rα2 expression in unstimulated cells and cytokine pre-treated cells. C: HASM cells were incubated with vehicle, IL-4 (5 ng/ml), IL-13 (5 ng/ml) or IFN-γ (10 ng/ml) for 24 hours. Percentage of positive cells in unstimulated cells and cells treated with IL-4, IL-13 and IFN-γ were assessed by flow cytometry and data presented as means ± SEM for 9 independent experiments * p < 0.05 compared to unstimulated cells (ANOVA).

To investigate whether changes in receptor expression could account for the inhibitory effects of cytokine pre-treatment on STAT6 phosphorylation induced by IL-13, HASM cells were treated with IL-4, IL-13 or IFN-γ for varying time periods and mRNA expression of IL-13 receptor subunits was assessed by real-time RT-PCR. As shown in Figure 4, IL-13Rα1 and IL-4Rα mRNA expression was unaffected by cytokine treatment. Both IL-4 and IL-13 increased expression of IL-13Rα2 mRNA by 3 to 4 fold over control but IFN-γ had no effect. The effects of cytokine pre-treatment on IL-13 receptor subunits mRNA expression were associated with changes in surface expression of receptor subunits using flow cytometry. HASM cells demonstrated low resting cell surface expression of all IL-13 subunits. As observed at the mRNA level, IL-4, IL-13 or IFN-γ did not alter the expression of the IL-13Rα1 (mean fluorescence intensity: non-stimulated: 2.5 ± 0.2; IL-4-stimulated: 2.45 ± 0.1; IL-13-stimulated: 2.5 ± 0.1; IFN-γ-stimulated: 2.45 ± 0.1, n = 3) and IL-4Rα subunits (mean fluorescence intensity: non-stimulated: 3.15 ± 0.75; IL-4-stimulated: 3.0 ± 0.3; IL-13-stimulated: 2.95 ± 0.3; IFN-γ-stimulated: 3.1 ± 0.2, n = 3) indicating that cytokine pre-treatments did not induce downregulation of these subunits on the cell surface. However, IL-4 and IL-13, along with IFN-γ, significantly up-regulated IL-13Rα2 cell surface expression in HASM cells as determined by both the percentage of positive cells (Figure 5) and the mean fluorescence intensity (non-stimulated: 7.26 ± 2.52; IL-4-stimulated: 25.63 ± 10.09; IL-13-stimulated: 24.82 ± 7.96; IFN-γ-stimulated: 22.43 ± 4.42, p < 0.05; Figure 5). All three cytokines significantly increased IL-13Rα2 expression at concentrations as low as 1 ng/ml (data not shown).

**Figure 6:**
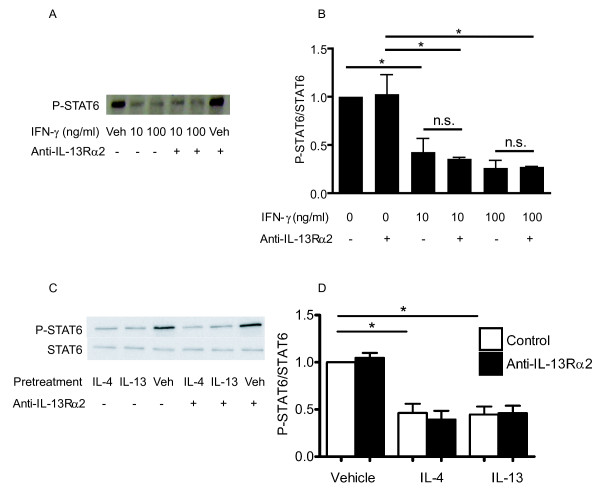
**Effect of blocking IL-13α2 on cytokine-mediated inhibition of IL-13-induced STAT6 phosphorylation**. HASM were pre-treated with IFN-γ (10 or 100 ng/ml), IL-4 (5 ng/ml) or IL-13 (5 ng/ml) 24 hours prior to IL-13 stimulation in the presence or absence of neutralizing concentration of anti-IL-13α2 polyclonal antibody (5 μg/ml). Phosphorylated STAT6 expression was assessed by Western analysis. A and C: Representative Western blots. B and D: Band densities were measured and the densitometric values were normalized to the IL-13-stimulated value. Data are shown as the mean values ± SEM of three independent experiments. * p < 0.05 (ANOVA).

Since the reduction of IL-13-induced STAT6 phosphorylation following IL-4, IL-13 or IFN-γ pre-treatment was associated with increased expression of IL-13Rα2, we investigated whether blocking the interaction between IL-13 and this subunit could restore the IL-13-induced STAT6 response. To this end, HASM cells were pre- treated with either IL-4, IL-13 or IFN-γ for 24 hours prior to IL-13 stimulation in the presence or absence of a neutralizing concentration of anti-IL-13Rα2 polyclonal antibody (5 μg/ml) added to cell cultures 1 hour prior to stimulation with IL-13. The antibody had no effect on IL-13- induced STAT6 phosphorylation *per se*. Figure 6 shows that the inhibitory effect of pre-treatment with either cytokine on IL-13 induced STAT6 phosphorylation was not altered by the addition of anti-IL-13Rα2 antibody used at a concentration that blocks 50% of the binding of 100 ng/ml of rhIL-13 to immobilized rhIL-13Rα2/Fc Chimera (a dose of IL-13 twenty times in excess of the concentration used in our study), according to the manufacturer's specifications. This antibody used at a slightly lower dose (4 μg/ml) has been shown to be effective in blocking IL-13Rα2-mediated effects on HASM [32].

### IL-4, IL-13 and IFN-γ upregulate SOCS-1 but not SOCS-3 mRNA expression in HASM cells

**Figure 7:**
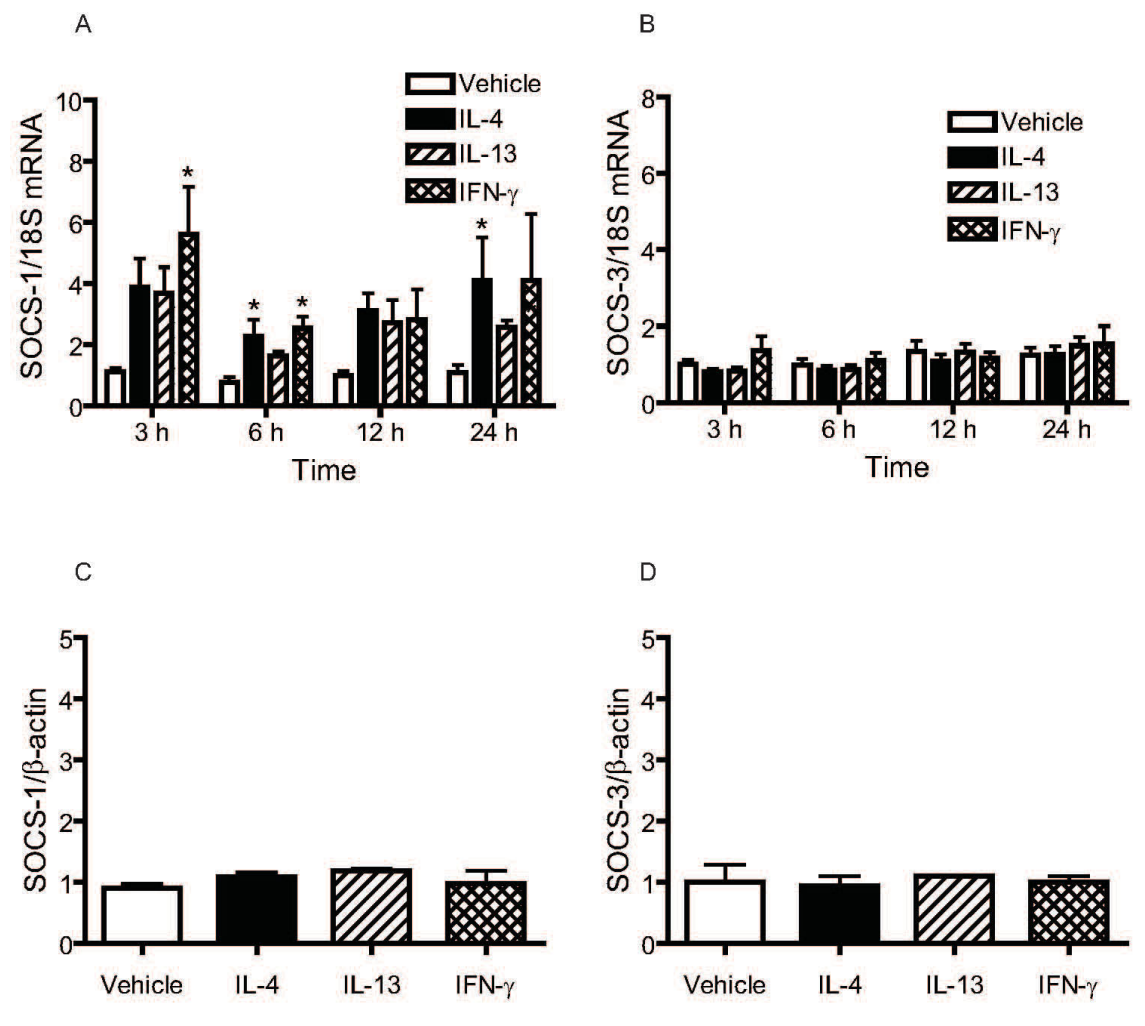
**Time-dependent effects of IL-4, IL-13 and IFN-γ on SOCS-1 (A) and SOCS-3 (B) mRNA and protein expression**. HASM were incubated with IL-4 (5 ng/ml), IL-13 (5 ng/ml) or IFN-γ (10 ng/ml) for 3, 6, 12 and 24 hours. SOCS-1, SOCS-3 and 18S mRNA expression was quantitated using real-time PCR. Data are expressed as the relative expression in SOCS/18S mRNA ratio and compared to unstimulated (vehicle) value of 1 at time 0, for 5 independent experiments. * p < 0.05 compared to cells treated with vehicle (ANOVA). SOCS-1 (C) and SOCS-3 (D) protein expression was assessed by Western analysis. Data are presented as mean ± SEM of densitometric values normalized to β-actin of 3–5 independent experiments.

SOCS-1 and SOCS-3 are members of the family of suppressors of cytokine signalling (SOCS) proteins involved in the negative regulation of signalling induced by IL-13 and IL-4. To investigate whether the inhibitory effects of IL-4, IL-13 and IFN-γ pre-treatment on STAT6 phosphorylation induced by IL-13 may be related to the induction of such proteins, the effects of IL-4, IL-13 and IFN-γ on the expression of SOCS-1 and SOCS-3 were determined at the mRNA and protein levels. IFN-γ and IL-4 significantly upregulated mRNA expression of SOCS-1 but had no effect on SOCS-3 mRNA expression (Figure 7). The increase in the expression of SOCS-1 mRNA following IL-13 stimulation did not reach statistical significance. Despite induction of SOCS-1 mRNA, we did not detect a significant change in protein expression by Western blot analysis.

### Effects of cytokine pre-treatment on IL-13-induced eotaxin release

**Figure 8:**
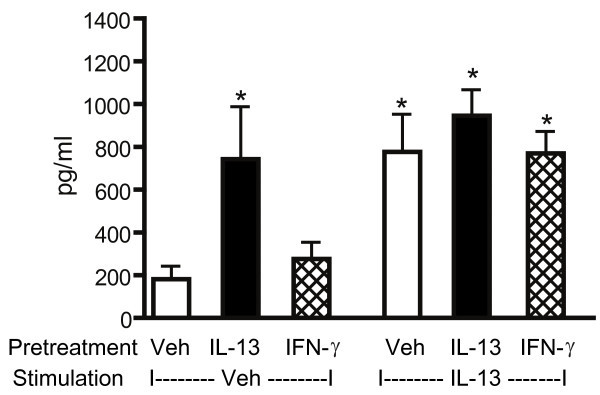
**Effects of cytokine pre-treatment on IL-13-induced eotaxin release**. HASM were pre-treated with appropriate vehicle (veh), IL-13 (5 ng/ml) or IFN-γ (10 ng/ml) for 24 hours followed by 24 hours stimulation with vehicle or IL-13 (5 ng/ml). Eotaxin levels in cell culture supernatants were assessed by ELISA. Data are presented as mean ± SEM of five independent experiments. * p < 0.05 compared to cells pre-treated with vehicle and stimulated with vehicle (ANOVA).

To assess the impact of IL-4, IL-13 and IFN-γ pre-treatment on a STAT6-dependent physiological outcome [29,33,34], we measured eotaxin release (Figure 8). Preliminary experiments revealed that significant but submaximal eotaxin release was observed when HASM cells were stimulated with IL-13 at a concentration of 5 ng/ml as compared to a higher concentration (5 ng/ml: 1040 ± 135 pg/ml; 10 ng/ml:1703 ± 180 pg/ml, p < 0.05). Dose-response experiments using IL-4 (1–20 ng/ml) indicated that significant eotaxin release occurred when cell were stimulated with IL-4 at doses ranging from 1 to 5 ng/ml and that no further increase in eotaxin release occurred with higher doses. Therefore, we could not evaluate the effects of IL-4 pre-treatment on IL-13-induced eotaxin production using our protocol since a maximal eotaxin production was reached. Pre-treatment with IL-13 resulted in significant eotaxin release. Subsequent stimulation with IL-13 for 24 hrs did not produce any further increase in eotaxin levels in cell culture supernatants suggesting that pre-treatment with IL-13 induced complete functional desensitization of the IL-13 receptor. Pre-treatment with IFN-γ did not alter eotaxin production on its own and did not inhibit IL-13-induced eotaxin production.

### IL-4, IL-13 and IFN-γ inhibit IL-13-mediated calcium responses to histamine

**Figure 9:**
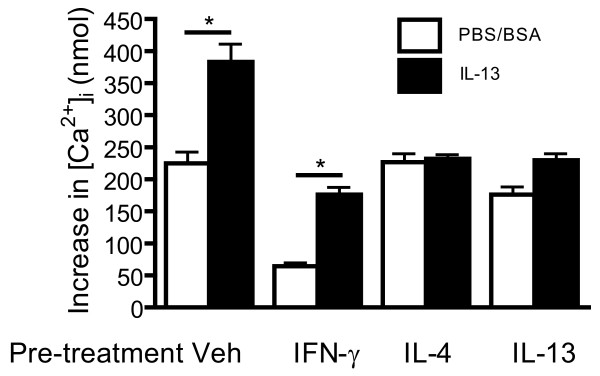
**Effects of cytokine pre-treatment on IL-13 augmented calcium response to histamine**. HASM were incubated with an appropriate vehicle or IFN-γ (10 ng/ml), IL-4 (5 ng/ml) or IL-13 (5 ng/ml) for 24 hours, followed by 24 hours incubation with vehicle (PBS/BSA) or IL-13. Calcium transients were measured in cells stimulated with histamine (1 μM). Data are expressed as calcium increase over baseline levels and presented as mean ± SEM of three independent experiments. *p < 0.05 compared to values obtained in cells incubated with PBS/BSA.

We next investigated whether cytokine pre-treatment modulates the effects of IL-13 on contractile responses of HASM cells to histamine. IL-13 enhanced the calcium response to histamine (Figure 9). Our results show that IL-4 and IL-13 pre-treatment abrogated the enhancement of calcium response to histamine in the presence of IL-13, again demonstrating the induction of desensitization to IL-13. The effects of IFN-γ differed to those of IL-4 and IL-13. Whereas IFN-γ reduced calcium responses to histamine both in the absence and the presence of IL-13 it did not abrogate the enhancement of the response to histamine by IL-13.

## Discussion

IL-13 has diverse effects on HASM cells that may have important implications for the pathobiology of asthma. However the responses of HASM cells to the presence of IL-13 is likely to be conditioned by the prevalent cytokine milieu. In this study, we sought to determine how cytokines representative of both the Th1 and Th2 types influence the responses of HASM cells to IL-13. The principal finding from our study is that the prototypical Th1 cytokine IFN-γ and the Th-2 cytokines IL-4 and IL-13 have different, although non-opposite, modulatory effects on IL-13 responses in HASM cells. All three cytokines induced the IL-13Rα2 subunit of the receptor while leaving the other subunits unaltered. IL-4, IL-13 and IFN-γ pre-treatment reduced IL-13 induced STAT6 phosphorylation, although the effect was most pronounced for IL-4 and IL-13. IL-13 pre-treatment abrogated IL-13 induced eotaxin secretion by HASM cells and also the IL-13-induced augmentation of the calcium transient evoked by histamine. Consistent with the lesser effect of IFN-γ pre-treatment on STAT6 phosphorylation in response to IL-13 there was also no significant reduction in eotaxin release or IL-13-mediated augmentation of calcium response to histamine.

The effects of Th1 and Th2 cytokines have been studied on a number of structural cells of the airways. IFN-γ has been shown to antagonize IL-13-induced STAT6 activity in airway epithelial cells, an effect that is time- and dose-dependent [35]. The inhibitory effect of IFN-γ on IL-4 signalling has been more extensively studied and has been demonstrated in both hematopoietic and non-hematopoietic cells including monocytes, lymphocytes, fibroblasts and airway epithelial cells [36-39]. Exposure of HASM cells to IFN-γ reduced the effects of IL-13 on STAT6 activation but did not appear to have much effect on calcium responses to histamine and on eotaxin secretion, important measures of contractile and secretory properties of HASM. Our data also point to a restriction of signalling through IL-13 in the presence of continuous amounts of IL-13 or IL-4. The desensitization induced by IL-4 and IL-13 was substantial and was sufficient to completely abrogate IL-13-induced effects. This may represent a negative feedback system by which these Th2 cytokines prevent persistence of IL-13 signalling at sites of allergic inflammation. Since these experiments have been performed using airway smooth muscle cells recovered from non-asthmatic subjects, the results of this investigation cannot be extrapolated to asthmatic airway smooth muscle cells. Whether these observed effects occur *in vivo *in normal or asthmatic airways also remains to be elucidated.

We examined some of the potential mechanisms by which desensitization of HASM cells to IL-13 may have occurred. First we addressed the effects of cytokine treatments on IL-13 receptor subunit expression. The mRNA and cell surface expression of the IL-13Rα1 and IL-4Rα subunits were unaffected by cytokine treatment suggesting that cytokine pre-treatment did not induce downregulation of synthesis or internalization of these subunits. These data are consistent with findings by Hirst et al. who demonstrated that both IL-4 and IL-13 do not alter IL-4Rα receptor expression in HASM cells [34], an effect that differs to that seen in murine smooth muscle cells [32], and in various immune cells [40,41]. Based on our findings, we can conclude that the inhibitory effect of all three cytokines on STAT6 activation is not mediated by altered cell surface expression of the IL-13Rα1 and IL-4Rα subunits. However, we observed an increased surface expression of IL-13Rα2, as evidenced by both the relative receptor density and the fraction of cells bearing IL-13Rα2. These data confirm and extend previous observations showing increased mRNA and cell surface expression of IL-13Rα2 in human [42] and murine ASM cells upon IL-13 stimulation. Likewise, lung levels of IL-13Rα2 mRNA are augmented in transgenic mice overexpressing IL-4, IL-13 or IFN-γ [43]. Interestingly, our data indicate that IFN-γ, unlike IL-4 and IL-13, did not increase IL-13Rα2 mRNA expression so that the increased cell surface expression of this receptor subset must be mediated by post-transcriptional or post-translational mechanisms. Indeed, IFN-γ induces the mobilization of IL-13Rα2 from intracellular pools to the cell surface in monocytes [20]. Regulation of IL-13Rα2 expression in HASM cells appears to slightly differ from those obtained in airway epithelial cells in which reduced IL-13 or IL-4 signalling induced by pretreatment with either IL-4 or IL-13 is associated with increase in expression of IL-13Rα2 mRNA and intracellular protein expression but no increase in cell surface receptor protein expression [43,44]. Likewise, in airway epithelial cells, IFN-γ induces IL-13Rα2 mRNA expression that was not associated with increased cell surface expression [37].

The IL-13Rα2 subunit may serve as either a decoy receptor in some circumstances [17-20] or as a signalling receptor in other situations [21]. Transfection of IL-13Rα2 into the airway epithelial cell line BEAS-2B led to downregulation of STAT6 phosphorylation [44], suggesting a role for this subunit in regulating cytokine signalling. Sparse data exist on the regulatory role of the IL-13Rα2 subunit in IL-13 responses in HASM. Using a blocking anti-IL-13Rα2 antibody Kellner et al have shown that this subunit likely modulates IL-13 responses as indicated by the reversal of decreases in HASM cell proliferation and acetylcholine-induced calcium mobilization in presence of high concentrations of IL-13 and IL-4 [32]. We used the same blocking antibody at a slightly higher concentration to assess the involvement of IL-13Rα2 on cytokine treatment effects on STAT6 phosphorylation. There was no demonstrable effect of the blocking antibody on the reduction in STAT6 activation, so that it is unlikely that upregulation of the expression of IL-13Rα2 accounts for the observation. These results are at variance with those reported by Kellner et al and may be related to differences in both the protocol and the functional outcomes studied. The lower concentrations of IL-13 and IL-4 used in our study may be more physiological than those used in Kellner's study. In other cell types, IL-13Rα2 inhibits IL-13 signalling in proportion to its level of expression and that these inhibitory effects could be overcome by high concentrations of IL-13 [45]. We cannot exclude the possibility that IL-13, although used at a concentration which did not induce maximal STAT 6 phosphorylation, was still present in sufficient amount in our experimental system to overcome the effects of the anti-IL-13Rα2 antibody. However in support of the concept that induction of IL-13Rα2 may not be the predominant regulator of cytokine signalling in HASM cells, we found that IFN-γ increased IL-13Rα2 expression slightly more than IL-4 or IL-13 yet was less effective at reducing IL-13 signalling as assessed by STAT6 phosphorylation.

We then tested whether the inhibition of phosphorylation of STAT6 was related to the induction of the endogenous inhibitory proteins, suppressors of cytokine signaling (SOCS) proteins. SOCS are inducible inhibitors, primarily in signaling cascades associated with the Jak-STAT pathway [46]. A variety of studies support this rationale. Over-expression of SOCS-1 and SOCS-3 in the human embryonic kidney HEK293 cells inhibit IL-4 and IL-13 mediated expression of eotaxin [47]. IFN-γ induces SOCS-1 and SOCS-3 in airway epithelial cells parallel to the inhibition of IL-4 signaling [37]. Both IFN-γ and IL-4 induce SOCS-1 gene expression in monocytes and macrophages, an effect that is associated with reduced activation of STAT6 by IL-4 [36,48]. IFN-γ-induces SOCS-1 and downregulates STAT6-dependent eotaxin production in murine fibroblasts [38]. Our data show that IL-4, IL-13 and IFN-γ all increased SOCS-1, but not SOCS-3, mRNA expression in HASM cells. The magnitude of induction of SOCS-1 mRNA was, however, substantially less than that reported for airway epithelial cells [37]. Despite this increase in SOCS-1 mRNA expression, there was no significant increase in protein expression. It is therefore unlikely that induction of SOCS molecules contributed significantly to the cytokine inhibition of IL-13-induced responses.

## Conclusion

In conclusion, IL-13 has effects on eotaxin secretion by HASM cells and calcium responses of HASM cells to histamine that are abolished by pre-treatment with IL-13. Pre-treatment with IL-4 also reduced IL-13 receptor signalling and calcium responses. A reduction in STAT6 phosphorylation in response to IL-13 was also found after treatment of the cells with IFN-γ but there was no evidence of desensitization of the HASM cells as measured by eotaxin secretion and calcium transients to histamine. The mechanism of IL-4 and IL-13 induced desensitization is not clear and does not appear to involve either induction of the IL-13Rα2 or the SOCS proteins. The observed desensitization to IL-13 may be a mechanism for limiting IL-13 induced responses in the airways in the setting of allergic airway inflammation.

## Abbreviations

HASM: human airway smooth muscle; STAT6: signal transducer and activator of transcription 6; AHR: airway hyperresponsiveness; JAK: Janus activated kinase; SOCS: suppressor of cytokine signalling.

## Competing interests

The authors declare that they have no competing interests.

## Authors' contributions

BM carried out Western blots and participated in the design of the study and preparation of the manuscript. BT and MM carried out the calcium microscopy. SEB performed the eotaxin measurements by ELISA. PF provided the samples for culture of the HASM. JM and SL conceived the study and were involved in drafting the manuscript. All authors read and approved the final manuscript.
